# Strategy for Withdrawal of Pharmacological Treatment for Urinary Incontinence in Children (StayDry): Protocol for an Open-Label Prospective Randomized Trial

**DOI:** 10.2196/63226

**Published:** 2025-07-09

**Authors:** Ann-Kristine Mandøe Svendsen, Søren Hagstrøm, Konstantinos Kamperis, Anna Elizabeth Andersen, Nanna Celina Henneberg, Jason Van Batavia, Anne Estrup Olesen, Luise Borch

**Affiliations:** 1 Department of Pediatrics and Adolescent Medicine Gødstrup Hospital Herning Denmark; 2 NIDO Centre for Research and Education Gødstrup Hospital Herning Denmark; 3 Department of Clinical Medicine Aarhus University Aarhus Denmark; 4 Department of Pediatrics and Adolescent Medicine Aalborg University Hospital Aalborg Denmark; 5 Department of Clinical Medicine Aalborg University Aalborg Denmark; 6 Department of Pediatrics and Adolescent Medicine Aarhus University Hospital Aarhus Denmark; 7 Department of Health Science and Technology Faculty of Medicine Aalborg University Aalborg Denmark; 8 Division of Pediatric Urology The Children's Hospital of Philadelphia Philadelphia, PA United States; 9 Clinical Pharmacology Aalborg University Hospital Aalborg Denmark

**Keywords:** urinary incontinence, withdrawal, children, solifenacin, mirabegron

## Abstract

**Background:**

To the best of our knowledge, no studies have investigated the withdrawal strategy of pharmacological treatment with solifenacin or mirabegron in children diagnosed with urinary incontinence who have achieved continence on pharmacotherapy.

**Objective:**

The primary objective is to investigate if abrupt withdrawal versus gradual withdrawal of pharmacotherapy (solifenacin or mirabegron) influences the risk of recurrence of incontinence, assessed by a self-reported 14-day calendar of incontinence episodes.

**Methods:**

Children aged 5-14 years diagnosed with urinary incontinence, treated with pharmacotherapy of solifenacin or mirabegron and ready for withdrawal, will be randomized 1:1 to either abrupt or gradual withdrawal, according to the medical treatment that the child is receiving. The primary outcome measure is the recurrence of incontinence after withdrawal, 1 month after initiation of withdrawal of the physician-prescribed medication, assessed by a self-reported 14-day calendar of incontinence episodes. In addition, recurrence of incontinence after 3, 6, and 12 months after initiation of withdrawal will be measured. The hypothesis that gradual withdrawal is superior to abrupt withdrawal regarding the risk of recurrence of incontinence will be analyzed by logistic regression.

**Results:**

Recruitment began at the end of May 2024 and will continue until 216 patients are included, which is expected by December 2027. As of February 2025, a total of 25 participants are included.

**Conclusions:**

The results are expected to influence the withdrawal strategy of pharmacological treatment with solifenacin or mirabegron in children with daytime urinary incontinence.

**Trial Registration:**

ClinicalTrials.gov NCT06465576; https://clinicaltrials.gov/search?term=NCT06465576

**International Registered Report Identifier (IRRID):**

DERR1-10.2196/63226

## Introduction

### Background

Urinary incontinence is a common condition, affecting up to 21% of children aged 5 to 7 years [1-4], and 4.5% of children and adolescents aged 11-16 years [3,4]. The most common cause of urinary incontinence is an overactive bladder [2,5]. The International Children’s Continence Society defines overactive bladder as a condition with urinary urgency with or without urinary incontinence among children and adolescents aged 5 years and older and without competitive pathology explaining the symptoms, such as neurogenic detrusor overactivity or urinary tract infection [6].

First-line treatment of urinary incontinence is urotherapy, aiming at improving the bladder reservoir function and voluntary bladder control [2]. If urotherapy is insufficient for achieving continence, second-line treatment in most clinical settings is the addition of pharmacological treatment aiming at suppressing bladder smooth muscle contraction [2].

In Denmark, the current stepwise pharmacological therapy consists of an antimuscarinic as solifenacin, or a beta3-adrenoreceptor agonist as mirabegron. If monotherapy of solifenacin or mirabegron is without effect, the child is treated with a combination of solifenacin and mirabegron. However, the pharmacological treatment with solifenacin and mirabegron of children with urinary incontinence is only sparsely investigated.

In children with daytime urinary incontinence, solifenacin and mirabegron have demonstrated efficacy in reducing daytime incontinence episodes [7-13]. Solifenacin and mirabegron are widely used off-label in the pediatric population, and only a few studies dealt with the efficacy and tolerability of the agents [7-10,14].

The pharmacological handling of pediatric incontinence is considered temporary, and withdrawal attempts are recommended after continence has been achieved [15]. There is no generally accepted withdrawal strategy for solifenacin or mirabegron in children. Currently, 2 different withdrawal strategies are being used in the clinical setting, namely abrupt withdrawal and gradual withdrawal, where the dosage prescribed is reduced or the interval between dosages given is increased.

Studies report on the differences in withdrawal strategies of antidiuretic therapy targeted at enuresis in pediatric populations, indicating that gradual withdrawal of desmopressin therapy results in prolonged intervals of continence without relapse [16-19]. One study has reported that gradual withdrawal of solifenacin is superior to abrupt withdrawal in adults [20]. However, to the best of our knowledge, no studies have investigated the strategy of withdrawal of pharmacological treatment with solifenacin or mirabegron in children diagnosed with urinary incontinence who have achieved continence on these pharmaceuticals.

Our study is proposed to ensure an evidence-based approach to a withdrawal strategy for pharmacological treatment with solifenacin or mirabegron in children with urinary incontinence.

### Objectives

The primary objective is to investigate if abrupt withdrawal versus gradual withdrawal of pharmacotherapy (solifenacin or mirabegron) influences the risk of recurrence of incontinence, assessed by a self-reported 14-day calendar of incontinence episodes. We hypothesize that gradual withdrawal is superior to abrupt withdrawal regarding the risk of recurrence of incontinence.

## Methods

### Trial Design

This is an open-label, prospective randomized trial, allocating participants to each of the 3 pharmaceutical groups, according to the physician-prescribed medical treatment that the child is receiving (solifenacin, mirabegron, or solifenacin in combination with mirabegron; Textbox 1). Within each pharmaceutical group, the participant will be randomized 1:1 to the intervention being compared: either abrupt withdrawal or gradual withdrawal. The study duration for each participant will be 12 months and encompasses 1 virtual meeting and 4 follow-up phone consultations.

**Overview of the pharmaceutical groups, according to the physician-prescribed medical treatment.**

**Solifenacin**

- 1A: abrupt withdrawal.
- 1B: gradual withdrawal.

**Mirabegron**

- 2A: abrupt withdrawal.
- 2B: gradual withdrawal.

**Solifenacin + mirabegron**

- 3A: abrupt withdrawal.
- 3B: gradual withdrawal.

### Eligibility Criteria

The inclusion and exclusion criteria for this study are provided in Textbox 2.

**Inclusion and exclusion criteria.**

**Inclusion criteria**

- The participants’ custody holders must voluntarily sign and date an informed consent before initiation of any study-specific procedures.
- Age 5 to 14 years old (inclusive) at the time of signing the consent and inclusion.
- Diagnosed with urinary incontinence as per The International Children’s Continence Society criteria.
- Pharmacological treatment with solifenacin or mirabegron.
- Continence has been achieved in pharmacological therapy with solifenacin or mirabegron.
- Previously, withdrawal attempts were accepted.
- Continence remained on the same dosage of medication for a minimum of 3 months.
- Continence remained for a minimum of 3 months (defined as urinary incontinence maximum 1 time a month or less during the last 3 months), self-reported by the participant and the participant’s custody holders.

**Exclusion criteria**

- The inability of the patents or parental custody holders to understand the Danish written and oral information.
- Neurogenic detrusor overactivity (neurogenic bladder).

### Intervention

Eligible children will be randomized 1:1 to either abrupt or gradual withdrawal of the physician-prescribed medication (solifenacin or mirabegron), the children have been treated with before entering the study. Abrupt withdrawal indicates stopping the prescribed medication on the date of withdrawal initiation. Gradual withdrawal involves taking the prescribed applied dosage of the medication every other day over 14 days, with the complete cessation of the medication occurring 14 days after the gradual withdrawal process begins.

### Withdrawal of Participants and Criteria of Discontinuation of Study

A study participant should be withdrawn from the study if at any time one of the following criteria applies:

1. Recurrence of incontinence during the course of the study (defined as >2 episodes of incontinence assessed by a 14-day calendar of incontinence episodes).
2. It is the wish of the participant (or their parents or parental custody holder[s]) for any reason (withdrawal of informed consent).
3. The discretion of the investigator is for safety reasons.
4. Severe compliance with study procedure as described in the protocol, as judged by the investigator.
5. The investigator judges it as necessary due to medical reasons.
6. Participants with a condition or a situation that, in the investigator’s opinion, may put the participant at significant risk or may interfere significantly with the participant’s participation in the study.
7. Participants with any other condition or situation that, in the investigator’s opinion, may be valid for discontinuation from the study.

### Primary Outcome

The primary outcome measure is the recurrence of incontinence after 1 month after initiation of withdrawal of the physician-prescribed medication, assessed by a self-reported 14-day calendar of incontinence episodes. In addition, recurrence of incontinence after 3, 6, and 12 months after initiation of withdrawal will be measured.

### Secondary Outcome

Secondary outcome measures are as follows. Development of symptoms occurring after abrupt withdrawal of the prescribed medication (solifenacin or mirabegron), assessed by self-reported questionnaires on listed symptoms, as a change from baseline up to 30 days after initiation of withdrawal. Development of symptoms occurring after gradual withdrawal of the prescribed medication (solifenacin or mirabegron), assessed by self-reported questionnaires on listed symptoms, as the change from baseline up to 44 days after initiation of withdrawal. Refer to the Multimedia Appendix 1 for the listed symptoms. The questionnaires are constructed by the authors and do not validate or apply CHERRIES (Checklist for Reporting Results of Internet E-Surveys) items.

### Procedures During the Trial

The participant timeline is illustrated in Table 1.

**Table 1:** Schedule of assessment.

Visit	Visit 1 (baseline)	Visit 2	Visit 3	Visit 4	Visit 5 (end of study)
Day	0	30	91	182	365
Visit schedule window	—^a^	±5 days	±5 days	±5 days	±5 days
Type of consultation	Virtual	Phone call	Phone call	Phone call	Phone call
Informed consent	✓	—	—	—	—
Inclusion and exclusion criteria	✓	—	—	—	—
Background information incl. demographics	✓	—	—	—	—
Randomization	✓	—	—	—	—
14-day calendar with incontinence episodes	—	✓	✓	✓	✓

^a^Not applicable.

### Recruitment

The participants will be included in outpatient incontinence clinics at the Department of Pediatrics at Aarhus University Hospital, Aalborg University Hospital, Regional Hospital Kolding, Regional Hospital Esbjerg, and Gødstrup Hospital.

Doctors and nurses specializing in urinary incontinence in the involved departments will be asked to screen patients. Possible eligible participants will be carefully informed orally and in writing about the objectives of the study, possible risks, and the study procedures, and answer questions regarding the study. Doctors and nurses employed at the involved outpatient clinics will call or send a direct note through the electronic patient journal to the coordinating investigator, site investigator, or his or her representative (a nurse with a specialty in childhood incontinence, affiliated with the involved pediatric departments and trained in the protocol).

Possible eligible participants and their parents or legal guardians will be contacted directly by the coordinating investigator, the site investigator, or his or her representative. The procedure for obtaining informed consent is elaborated in the Informed Consent subsection*.*

The site investigator or his or her representative must ensure eligibility. If a possible participant and their parents or legal guardians are willing to participate after the relevant time for consideration, they are invited to visit 1 in the outpatient clinic or by phone or via virtual contact. Here, informed oral and written consent from the parents or legal guardians will be obtained and signed and dated by the parents or legal guardians and an investigator.

### Visit 1

Participants will enter the study after providing informed consent by parents or parental custody holders. Background information (including age, gender, height, weight, urinary incontinence history, any other medical diagnosis, including psychiatric diagnosis, any comedication, or any withdrawal of comedication) will be obtained.

The participant will be allocated to each of the 3 pharmaceutical groups, according to the physician-prescribed medical treatment that the child is receiving. Within each pharmaceutical group, the participant will be randomized 1:1 to the intervention being compared: abrupt withdrawal or gradual withdrawal. The date of initiation of withdrawal of pharmacotherapy will be decided.

### Visits 2, 3, 4, and 5

Follow-up phone consultations with the coordinating investigator or the site investigator are planned 1, 3, 6, and 12 months following initiation of withdrawal. Data collected at the follow-up visits are the status of continence. The participant will fill out a 14-day calendar of incontinent episodes before each visit. Recurrence of incontinence (defined as >2 episodes of incontinence assessed by a self-reported 14-day calendar of incontinence episodes) or no recurrence of incontinence will be registered.

### Questionnaire on Withdrawal Symptoms

**Figure 1:**
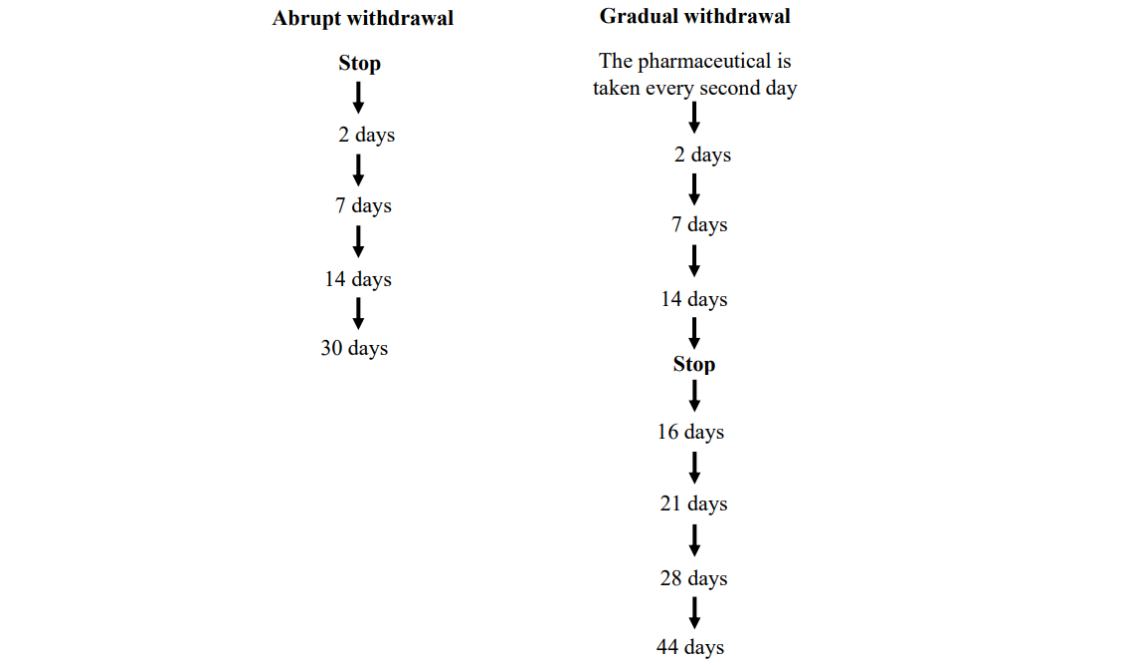
A detailed description and timeframe of how questionnaires on withdrawal symptoms should be answered regarding the applied withdrawal method.

During the study period, participants will be instructed to complete electronic questionnaires regarding withdrawal symptoms associated with the withdrawal of medication. The number of times and when the participant must complete the questionnaire is dependent on the allocated withdrawal method (Figure 1). The questionnaires can be completed with assistance from the parents.

### Randomization Procedure

Randomization will be performed in the REDCap (Research Electronic Data Capture, Vanderbilt University) portal and will occur centrally. An electronic case report form is built for the study data to be entered and for the randomization to be performed. Following written informed consent, randomization is stratified by a 1:1 allocation within each stratum using predefined block sizes for sites. Randomization is by a computer-generated random number list.

### Statistical Analysis

Continuous variables will be presented as median with interquartile range for nonnormal distributed data, and as mean (SD) for normally distributed data. Categorical data will be presented as numbers and percentages. The distribution of data will be analyzed using histograms and QQ plots.

The hypothesis that gradual withdrawal is superior to abrupt withdrawal regarding the risk of recurrence of incontinence will be analyzed by logistic regression.

Two logistic regression models with different variations of adjustment. One logistic regression model with adjustment for each prescribed medication, as the randomization stratifies within the prescribed medication group. Another logistic regression model in which variables are adjusted due to suspicion of possible effect modification. The degree of adjustment will follow the event per the Variable rule. The logistic regression models will be analyzed at data of recurrence or not after 1 month, and hereafter 3, 6, and 12 months for a decreasing population who remain. Participants with recurrence of urinary incontinence will be described with descriptive statistics. The secondary outcome of side effects related to withdrawal will be presented with descriptive statistics.

### Sample Size Calculation

To our knowledge, there is no available data regarding the efficacy of withdrawal strategies for pharmacological treatment with solifenacin or mirabegron in children diagnosed with urinary incontinence. Few studies have evaluated desmopressin withdrawal in children with enuresis. In the lack of alternatives, we have based our sample size calculation on these available desmopressin data, assuming a relapse rate of 40% with abrupt withdrawal of pharmacological treatment and 10% with gradual withdrawal of pharmacological treatment. With a significant level of .05 and a power of 80%, the minimal required sample size for adequate power is 64 participants in each group. We expect a maximum dropout rate of 10%. Therefore, 72 participants are included in the groups, for a total of 216 participants.

### Ethical Considerations

#### Overview

The study is authorized by the authorities of the Danish Medicines Agency and the Danish Health Research Ethics Committee and registered at Clinical Trials in the European Union (EU CT 2023-510280-35-00). To our knowledge, no studies have been performed investigating the potential outcomes of abrupt versus gradual withdrawal of solifenacin or mirabegron used by children diagnosed with urinary incontinence. To our knowledge, no literature has assessed the connection between the abrupt withdrawal of antimuscarinic drugs and the development of withdrawal syndrome in children with urinary incontinence.

All pharmacological side effects will be handled in accordance with Danish legislation. No risk or unknown side effects are expected from medical treatment or withdrawal. The therapeutic potential for future patients justifies the project to be carried out. Participation in this study will not lead to any disadvantages for the patient in their treatment.

The study will be conducted in accordance with the protocol, applicable regulatory requirements according to Good Clinical Practice, and the ethical principles of the Declaration of Helsinki. Information regarding the participants is protected and anonymized according to the General Data Protection Regulation and the actual law. The study participants and their families will not be economically compensated for participating in the study.

#### Informed Consent

As a part of the process of obtaining informed consent, it is considered essential that face-to-face communication takes place. If the first visit is a virtual meeting, it will take place using Central Denmark Region’s virtual meeting application. The virtual meeting application is encrypted to protect the confidential information that will be discussed.

The investigator will carefully explain orally the objectives of the study, possible risks, and the study procedures, and answer questions regarding the study. It must be ensured by the investigator that the possible participant or the parents have understood the information, and that informed consent is obtained. The possible participant should be informed within the limits of his or her understanding and with age-appropriate words.

If a possible participant and parents or parental custody holders are willing to participate, informed oral and written consent from the parent or parental custody holders will be obtained, signed, and dated by the parents or parental custody holders and the investigator. In case of a virtual visit, the informed consent is signed electronically.

#### Safety

Safety evaluation includes registration of adverse events and adverse reactions, as well as serious adverse events and reactions.

#### Registration

The trial is in accordance with the CONSORT (Consolidated Standards of Reporting Trials) checklist. The study is registered at the research inventory of the Regions of Denmark (1-16-02-211-24) and Aarhus University (ARG-2024-731-23833). The results will be submitted to Clinical Trials in the European Union within 1 year after the end of the trial. Furthermore, data will be published in the Clinical Trials Register.

## Results

The StayDry study was approved by Clinical Trials in the European Union on May 22, 2024. Recruitment began on May 27, 2024, and will continue until 216 patients are included, which is expected by December 2027. As of February 2025, a total of 25 participants are included. The results will be submitted to Clinical Trials in the European Union and ClinicalTrials.gov within 1 year after the end of the trial.

## Discussion

### Anticipated Findings and Previous Work

This study is designed to provide valuable insights into the optimal withdrawal strategy for solifenacin and mirabegron in children with urinary incontinence, as this trial will fill a significant gap in the current literature.

The existing literature on pharmacological withdrawal in pediatric populations does not exist, particularly regarding antimuscarinics like solifenacin and mirabegron. More established literature exists on the withdrawal of desmopressin, a treatment used for enuresis. Studies examining the withdrawal of desmopressin therapy in children have found that gradual withdrawal is associated with a lower risk of relapse compared with abrupt cessation. Several studies have reported that gradual tapering of desmopressin results in longer periods of continence after discontinuation without relapse [17-19]. These findings have led to the widespread adoption of gradual withdrawal as the preferred approach for discontinuing desmopressin therapy.

Previous research in adult populations has focused on the effects of discontinuing antimuscarinics for overactive bladder [20], but findings may not be directly applicable to children. The trial has the potential to optimize the medical withdrawal and minimize the risk of recurrence of urinary incontinence after withdrawal of pharmacotherapy. The results are expected to influence the withdrawal strategy of children with urinary incontinence.

### Strengths and Limitations

The strength of our study is the randomized multicenter design, encompassing 5 pediatric departments across Denmark. This ensures broad applicability and relevance in daily clinical practice. The study is embedded into and reflects daily clinical practice. The pragmatic design ensures the timely inclusion of patients and excludes participants with relapses of urinary incontinence.

The main limitation is the lack of blinding, which could introduce bias by influencing outcome assessment or potentially introducing placebo effects. Without blinding, investigators and participants are aware of withdrawal allocation, which can lead to biased behavior while answering the questionnaires and data interpretation. Another limitation is no background knowledge about socioeconomic factors.

By conducting the study in real-life settings with medication that is familiar to patients and parents, the findings are more likely to apply to clinical practice, as they reflect the challenges and circumstances encountered in routine medical care. Furthermore, patients are more likely to adhere to treatment protocols involving medications they are familiar with or have easy access to through standard prescriptions, increasing the feasibility of recruitment and retention in the study.

### Conclusion

If gradual withdrawal is superior to abrupt withdrawal, the children with urinary incontinence treated with solifenacin or mirabegron will benefit significantly from this knowledge once children with urinary incontinence can start withdrawal medical treatment.

## Data Availability

The datasets generated during this study are planned to be available upon reasonable request.

## Appendix

Multimedia Appendix 1 Questionnaire on withdrawal symptoms.

## Abbreviations

CHERRIES: Checklist for Reporting Results of Internet E-Surveys
CONSORT: Consolidated Standards of Reporting Trials
REDCap: Research Electronic Data Capture
