# Supplementary material for: Strategy for Withdrawal of Pharmacological Treatment for Urinary Incontinence in Children (StayDry): Protocol for an Open-Label Prospective Randomized Trial
Source: JMIR Res Protoc. 2025 Jul 9;14:e63226. doi: 10.2196/63226 (PMC12287669; doi:10.2196/63226)
Supplement: Multimedia Appendix 1 [file resprot_v14i1e63226_app1.pdf]

Dear participant and parents,

Thank you for participating in the StayDry research project. As part of your participation in the project, we would like to ask you to answer this questionnaire. Please tick the statement that best suits you as a participant/your child based on the question.

| Have you since last had …? | Never | Sometimes | Often | Always |
|---|---|---|---|---|
| Symptoms that may result from treatment with med solifenacin and/or mirabegron | | | | |
| Dry mouth | | | | |
| Blurred vision/difficulty seeing clearly | | | | |
| Discomfort with bright light | | | | |
| Constipation | | | | |
| Difficulty urinating | | | | |
| Heartbeating | | | | |
| Dizziness | | | | |
| Falling trend | | | | |
| Symptoms that may be due to withdrawal of solifenacin and/or mirabegron | | | | |
| Increased saliva flow | | | | |
| Nausea | | | | |
| Vomiting | | | | |
| Diarrhea | | | | |
| Urgency | | | | |
| Increased sweating | | | | |
| Headache | | | | |
| Sleep disorders | | | | |
| Vivid dreams | | | | |
| Nightmare | | | | |
| Restlessness | | | | |
| Anxiety | | | | |
| Been confused | | | | |
